# BRM: the core ATPase subunit of SWI/SNF chromatin-remodelling complex—a tumour suppressor or tumour-promoting factor?

**DOI:** 10.1186/s13072-019-0315-4

**Published:** 2019-11-13

**Authors:** Iga Jancewicz, Janusz A. Siedlecki, Tomasz J. Sarnowski, Elzbieta Sarnowska

**Affiliations:** 1 Department of Molecular and Translational Oncology, The Maria Sklodowska-Curie Institute-Oncology Center in Warsaw, Wawelska 15B, 02-034 Warsaw, Poland; 2 Institute of Biochemistry and Biophysics Polish Academy of Sciences, Pawinskiego 5A, 02-106 Warsaw, Poland

**Keywords:** BRM, *SMARCA2*, SWI/SNF chromatin-remodelling complex (CRC), Cancer, Epigenetics, Small molecule inhibitors, Synthetic lethality

## Abstract

BRM (BRAHMA) is a core, SWI2/SNF2-type ATPase subunit of SWI/SNF chromatin-remodelling complex (CRC) involved in various important regulatory processes including development. Mutations in *SMARCA2*, a BRM-encoding gene as well as overexpression or epigenetic silencing were found in various human diseases including cancer. Missense mutations in *SMARCA2* gene were recently connected with occurrence of Nicolaides–Baraitser genetics syndrome. By contrast, *SMARCA2* duplication rather than mutations is characteristic for Coffin–Siris syndrome. It is believed that BRM usually acts as a tumour suppressor or a tumour susceptibility gene. However, other studies provided evidence that BRM function may differ depending on the cancer type and the disease stage, where BRM may play a role in the disease progression. The existence of alternative splicing forms of *SMARCA2* gene, leading to appearance of truncated functional, loss of function or gain-of-function forms of BRM protein suggest a far more complicated mode of BRM-containing SWI/SNF CRCs actions. Therefore, the summary of recent knowledge regarding BRM alteration in various types of cancer and highlighting of differences and commonalities between BRM and BRG1, another SWI2/SNF2 type ATPase, will lead to better understanding of SWI/SNF CRCs function in cancer development/progression. BRM has been recently proposed as an attractive target for various anticancer therapies including the use of small molecule inhibitors, synthetic lethality induction or proteolysis-targeting chimera (PROTAC). However, such attempts have some limitations and may lead to severe side effects given the homology of BRM ATPase domain to other ATPases, as well as due to the tissue-specific appearance of BRM- and BRG1-containing SWI/SNF CRC classes. Thus, a better insight into BRM-containing SWI/SNF CRCs function in human tissues and cancers is clearly required to provide a solid basis for establishment of new safe anticancer therapies.

## Main text

### Background

The genomic DNA in eukaryotic cells nuclei is packaged together with histone proteins into a complex called chromatin, enabling the storage of a relatively large amount of DNA in a very compacted form. However, the structure of chromatin restricts the contact between DNA and various protein (e.g. activators, repressors, modifying enzymes) or non-protein (e.g. enhancers, silencers) regulatory elements. Therefore, chromatin is a target for various modifications including chromatin remodelling, which controls the access to DNA sequences. The process is executed by multiprotein chromatin-remodelling complexes (CRCs), which utilise energy from ATP hydrolysis [1].

### BRM ATPase, the SWI2/SNF2-type core subunit of SWI/SNF chromatin-remodelling complexes

One of the best-characterised chromatin-remodelling complexes (CRCs) are SWI/SNF CRCs. Originally, the SWI/SNF CRC was described in baker’s yeast, where it affected mating-type switch (SWI) [2] and sucrose fermentation (SNF—sucrose non-fermenting) [3, 4]. The SWI/SNF complexes were thus named for these phenotypic alterations [5]. Homologues of yeast SWI/SNF CRCs’ subunits were subsequently found in other organisms including humans [6], proving that they are highly evolutionary conserved among all Eukaryotes [1]. The SWI/SNF CRCs are involved in the regulation of various crucial cellular processes such as the cell cycle, cell morphology and adhesion, apoptosis, signal transduction, DNA repair and stress response, which are frequently and significantly altered in cancer [7–10].

SWI/SNF CRCs are multiprotein complexes, built from 10 to 15 subunits. Depending on the subunit composition, several classes of SWI/SNF CRCs may exist in the cell simultaneously [11]. The SWI/SNF subunit composition and activity is cell/tissue-specific [12].

It was believed that the core complexes of all types of SWI/SNF CRCs consist of four core subunits—one of two ATPase subunits: BRM (encoded by *SMARCA2 gene*) or BRG1 (encoded by *SMARCA4* gene), BAF155 (encoded by *SMARCC1* gene), BAF170 (encoded by *SMARCC2* gene) and INI1 (SNF5 or BAF47, encoded by *SMARCB1* gene) [7, 13]. Together with the core complex, non-core subunits are present in the SWI/SNF CRCs. The number of non-core subunits may differ and their composition influences activity of the whole complex.

Recent studies by several research groups indicated the existence of non-canonical SWI/SNF CRC classes lacking some core (i.e. INI1) and non-core subunits [14, 15]. Furthermore, the presence of non-canonical SWI/SNF complexes was also shown using mouse models [16].

The SWI/SNF CRCs utilise energy from ATP hydrolysis to disrupt contact between the DNA and histones, leading to nucleosome disassembly [17, 18]. They control gene expression by moving or removing nucleosomes covering binding sites for transcription factors [19] or stabilising nucleosome positions. The activity of SWI/SNF CRCs requires recruitment to the DNA by transcription regulators and other factors [20]. The action of SWI/SNF CRCs alters upon interactions with various proteins such as hormone receptors [21], acetylases/deacetylases, etc. and depends on the modification of its subunits by, e.g. acetylation, as has been observed for the BRM ATPase subunit. A comprehensive summary of SWI/SNF CRCs’ action was provided by Sarnowska et al. [19].

BRM and BRG1 ATPase subunits are critical for the SWI/SNF activity. Both of them belong to the SWI2/SNF2 family, share about 75% structural homology and share similar ATPase and helicase activities [6], although their function is not identical. In humans, BRG1 ATPase may be present in both SWI/SNF CRC classes—BAF (BRM or BRG1-associated factors) and PBAF (polybromo BRG1-associated factors), while BRM has been found in BAF class of SWI/SNF complexes only and is the so-called signature subunit of this complex class. BRM has lower ATPase activity than BRG1 [22, 23], therefore, its less important role was postulated.

This hypothesis has been supported by mouse models where *Brm*-knockout (*Brm*^−/−^) mice lived until adulthood and developed tumours while Brg1^−/−^ null mutants caused embryonic lethality [24]. It has also been shown that Brm controls cellular proliferation by regulation of the cell cycle [25]. The mouse model study revealed that both homozygous and heterozygous loss of *Brm* resulted in an increased risk of tumour development, when exposed to carcinogens [22]. Therefore, it is proposed that Brm rather acts as a cancer susceptibility than a tumour suppressor gene [26]. The importance of Brm in mice has been shown by several additional studies, i.e. using conditional knockout of both genes encoding BRM and BRG1 ATPases in heart. In this case, the concomitant depletion of Brm and Brg1 resulted in severe cardiac dysfunction associated with glycogen accumulation and mitochondrial defects, eventually leading to death [27]. Moreover, functional Brm protein is crucial for the initiation of regeneration phase after liver injury and dominates during the late injury phase on Brg1 function [28].

The double-knockout mice *Brm*^−/−^/Brg1^−/−^ exhibited an unexpected ability to overcome loss of both ATPases. In fact, *Brm*^−/−^/Brg1^−/−^ mice restored Brm expression via an alternative splicing strategy which resulted in production of truncated but functional Brm protein [29]. This study, together with the existence of alternative splicing variants of *SMARCA2* gene [30], indicate that the role of BRM may be more complicated than so far reported. It may be due to the fact that several different forms of BRM protein may exist in the cell.

The loss of human BRM or BRG1 consequently leads to the modified expression of genes that are significant for tumour development, e.g. genes encoding tumour suppressors. Many of them control cellular processes such as metabolism (including drug metabolism), DNA repair, differentiation, adhesion and apoptosis, and are involved in angiogenesis, progression or metastasis of cancer [31]. Recent studies on ovarian cancer revealed that BRG1 and BRM ATPases are mutually exclusive as their parallel inactivation leads to synthetic lethality [32], although other reports indicated the survival of cells with depletion of both ATPases [31]. One possible explanation of this apparent discrepancy may be the existence of shorter, truncated but functional versions of BRM protein which are simply not recognised by the anti-BRM antibody.

**Fig. 1:**
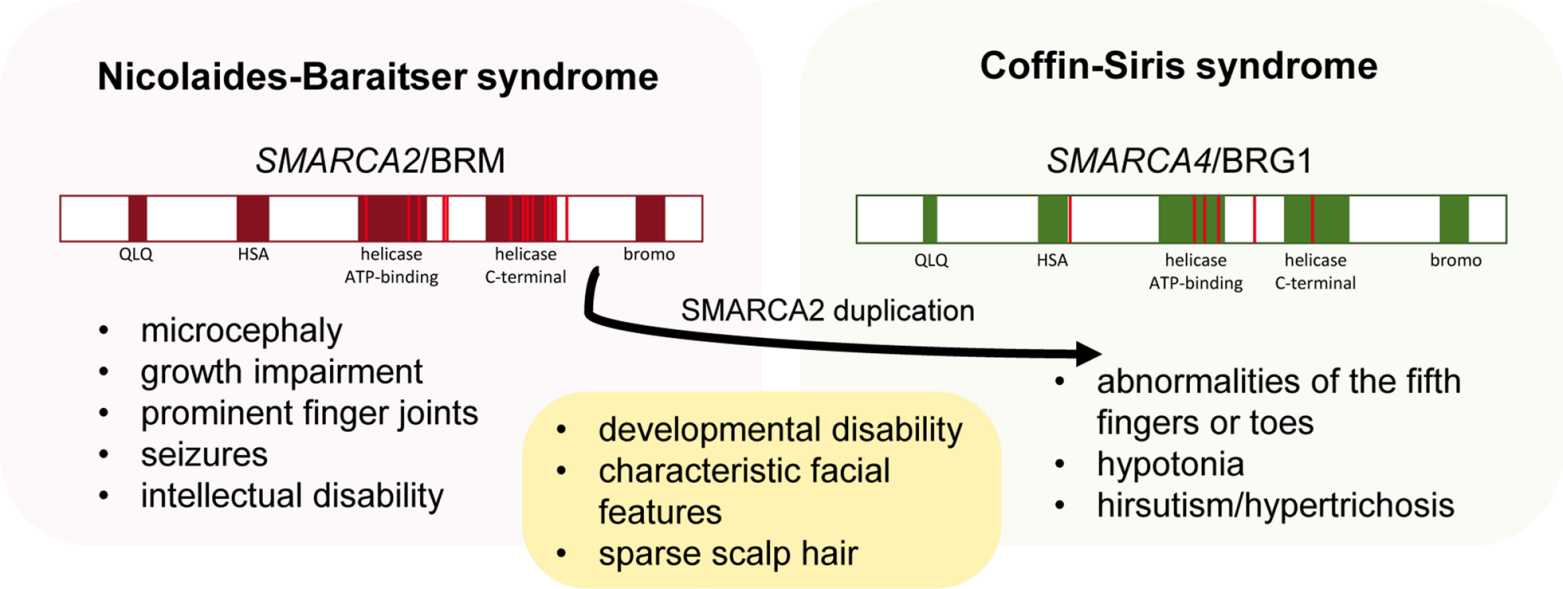
The phenotypic differences and commonalities between NCBRS and CSS. The red lines correspond to mutation sites in SMARCA2 and SMARCA4 genes according to [136]

In human heterozygous missense, mutations in BRM-encoding *SMARCA2* gene were identified in patients with Coffin–Siris (CSS) and Nicolaides–Baraitser (NCBRS) syndromes [33]. Although the last study performed on a large cohort of CSS patients proved that they carry the missense mutation in *SMARCA4* (BRG1 encoding gene) gene but not in BRM-encoding *SMARCA2* gene. In some CSS patients, duplication of the *SMARCA2* gene was detected [34]. On the other hand, missense mutations in *SMARCA2* were detected in NCBRS patients [35]. Moreover, one individual with *SMARCA2* mutation was previously diagnosed with CSS and after was reclassified for NCBRS [36, 37]. This collectively suggests that missense mutations in *SMARCA4* and *SMARCA2* may cause different developmental disabilities, although both CSS and NCBRS syndromes share some similar developmental dysfunctions and their distinction is based mostly on foot and hand features [35] (Fig. 1).

The occurrence of developmental alterations in CSS and NCBRS could be in contradiction with mouse models, where heterozygous loss in the Brm-encoding gene has no developmental effects. Although, in CSS the duplication of *SMARCA2* gene may lead to overexpression of BRM protein and as a consequence altered SWI/SNF stoichiometry caused by the pathological competition of BRM with BRG1 ATPase. In NCBRS, the missense mutations in *SMARCA2* may result in BRM gain-of-function or loss of function, i.e. by fast protein degradation. The exact role of *SMARCA2* missense mutations or duplication during development is still unclear; however, the *SMARCA2* polymorphisms may lead to higher cancer risk, suggesting the role of human BRM as a cancer susceptibility gene, similarly to mice [38].

It is also important to note that the classes of human SWI/SNF CRCs containing BRG1 or BRM subunits may regulate different promoters and sometimes they even differentially regulate transcription of the same genes [19]. This could be based on the differences in transcription factor recruitment, subunit composition and the occurrence of differential modifications of SWI/SNF subunits [23]. The picture of BRM/BRG1 interdependence is broadened by the in-depth, high throughput study based on ChIPseq and RNAseq analysis of BRM or BRG1-depleted HepG2 cell line. This study revealed that depletion of one ATPase subunit frequently leads to decreased abundance of the remaining subunit. Additionally, on numerous genes, the remaining subunit is either retained or gained [39]. All the above data suggest that in cancer, both ATPases have similar functions and could be partially redundant.

### Mechanism of action

BRM-containing SWI/SNF CRCs regulate expression of a large number of genes involved in carcinogenesis including (i) epithelial–mesenchymal transition genes, e.g. *CDH2* (N-cadherin) and SNAI1; (ii) cell cycle genes, e.g. *CCND1* (cyclin D1), *CCNE2* (cyclin E2), *CDK4* and *CDK6* (cyclin kinases), (iii) metabolic genes, e.g. *GAPDH*, *ALDOA* and *LDHA*; (iv) cancer suppressor genes and oncogenes, e.g. *BRCA1*, *PTEN*, *AKT1*, *HRAS* and *KRAS*. Importantly, BRM also regulates expression of self-encoding *SMARCA2* gene and other SWI/SNF subunits [40].

**Fig. 2:**
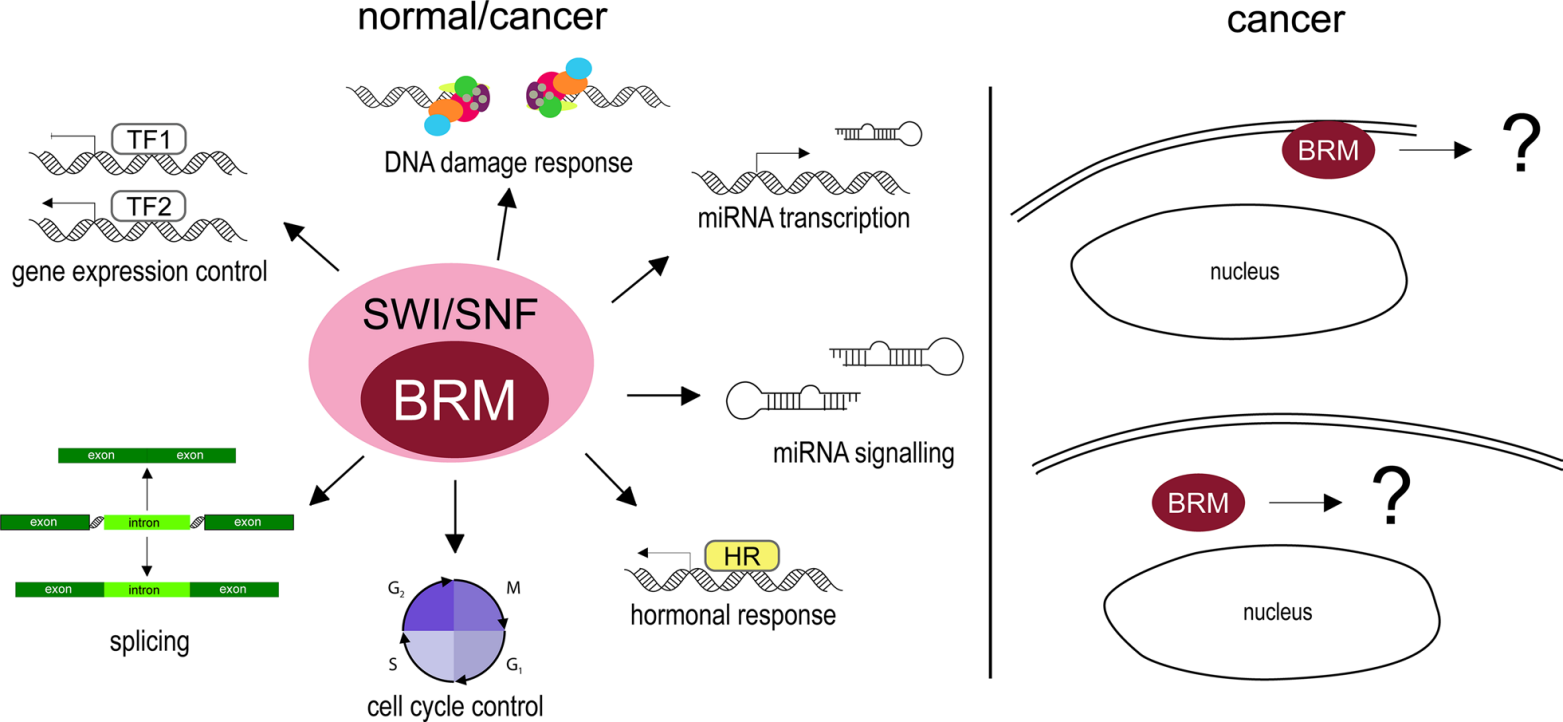
BRM-involving cellular processes. BRM protein is involved in a variety of cellular processes both in healthy/normal and cancerous cells; for example, gene expression control, alternative splicing, cell cycle control, participating in hormonal response and miRNA transcription and signalling. In pathological situations, in cancer cells, BRM can leave the cell nuclei and migrate to cytoplasm or cell membrane, although specific effects of BRM in these locations are unknown

BRM directly interacts with the retinoblastoma protein (Rb) and its family members [7, 41, 42]. Through this interaction, BRM influences cell cycle, causing repression of E2 promoter binding factor (E2F) family transcription factors [43]. Cells lacking BRM cannot enter the G_1_/S phase resulting in growth arrest [44] (Fig. 2). BRM function in the cell cycle is probably dependent on the phosphorylation of BRM by cyclin E/CDK2 complex causing dissociation of Rb from ATPase [45], and leading to cell cycle progression [46]. However, some data suggest that BRG1 has a more significant role in cell cycle control than BRM and, therefore, the specific role of BRM in Rb-mediated cell cycle inhibition remains elusive [47].

The link between SWI/SNF CRCs and TP53, a commonly mutated oncogene, is also known [48]; however, the particular role of BRM ATPase in this dependency is still unclear. Xu and colleagues demonstrated that BRM and BRG1 affect TP53-dependent p21 transcription differently. BRG1 knock-down handicaps TP53 binding to p21 promoter although BRM has ability to replace BRG1 in TP53 regulation [49].

Interestingly, mice lacking Brm did not present pathological *Tp53* mutations in tumours, although such mutations were accumulated in Brm-positive tumours, suggesting that loss of Brm would restrain selection of *Tp53*-mutated variant in tumour evolution. Collectively, this observation strongly suggests the existence of various ways of tumour evolution and development [47].

Some data indicate SWI/SNF participation in DNA damage response. BRM is involved in non-homologous end-joining (NHEJ) DNA repair, although its activity in this process depends on SWI/SNF complex composition [50]. BRM recruitment to double-strand breaks depends on, i.e. histone 2B phosphorylation on Ser36 which promotes BRM involvement in this process. Ribeiro-Silva and colleagues [51] observed that BRM is required for correct recruitment of the transcription factor II H (TFIIH) to the DNA damage site and facilitates DNA nucleotide excision repair pathway. Moreover, the SWI/SNF CRC also participates in DNA damage repair by interactions with BRCA1, indicating its important role in homologous recombination [52] (Fig. 2).

BRM also plays an important role in regulation of alternative splicing via interaction with spliceosome components. BRM overexpression favours inclusion of alternative exons [53], which is consistent with the observation that BRM is present not only on promoter regions, but also in the gene body [39] (Fig. 2).

Sakurai et al. [54] and Kobayashi et al. [55] demonstrated that BRM participates in the miRNA containing axis, particularly miR-199 and transcriptionally regulates the miR-302a-3p expression [56]. BRM is also involved in JAK2/STAT3 pathway, causing its activation [57]. The SWI/SNF BRM-containing CRC acts in the control of hormonal signalling pathways and participates in hormonal crosstalk [19]. In particular, BRM is required for proliferation of androgen-dependent prostate cancer [58] and regulates androgen receptor (AR) target genes expression [59]. Additionally, in cooperation with prohibitin, BRG1 or BRM ATPases are crucial for oestrogen antagonist-mediated breast cancer growth suppression [60]. The known BRM interactors and processes involving BRM are summarised in Table 1. The large interaction network of BRM suggests a strong potential impact of any BRM impairment on numerous regulatory processes.

**Table 1:** The known BRM interactors and processes involving BRM

Lp.	Full name	Abbreviations	Gene name(s)	Function; biological process	Refs.
Transcription factors					
1	Androgen receptor	AR	*AR*, *DHTR*, *NR3C4*	Transcription regulation, hormone receptor	[137–139]
2	Breast cancer type 1 susceptibility protein; breast cancer 1, early onset	BRCA1	*BRCA1*, *RNF53*	DNA repair, transcription regulation, metabolism	[140–142]
3	CCAAT/enhancer-binding protein alpha	C/EBP alpha, CEBPA	*CEBPA*, *CEBP*	Transcription regulation, DNA-binding	[143]
4	Chromobox protein homolog 5	CBX5, HP1 alpha	*CBX5*, *HP1A*	Transcription regulation, chromatin organisation	[144, 145]
5	Endothelial PAS domain-containing protein 1	EPAS-1, bHLHe73, HLF, HIF-2-alpha	*EPAS1*, *BHLHE73*, *HIF2A*, *MOP2*, *PASD2*	Transcription factor, hypoxia induced regulator of oxygen related genes	[146]
6	EP300-interacting inhibitor of differentiation 1	EP300, EID-1	*EID1* C15orf3, CRI1, RBP21, PNAS-22, PTD014	Transcription regulation, cell cycle, differentiation	[147]
7	Oestrogen receptor	ER	*ESR1*, *ESR*, *NR3A1*	Transcription regulation, hormone receptor	[148]
8	Histone H2A deubiquitinase MYSM1	2A-DUB, MYSM1	*MYSM1*, *KIAA1915*	Transcription regulation, chromatin regulator	[149]
9	Histone deacetylase 1	HD1, HDAC1	*HDAC1*, *RPD3L1*	Histone deacetylase, transcription regulation	[22, 150]
10	Histone deacetylase 2	HD2, HDAC2	*HDAC2*	Histone deacetylase, transcription regulation	[22, 150]
11	Histone-lysine N-methyltransferase EZH2	EXH2, ENX-1	*EZH2*, *KMT6*	PRC2 main subunit, transcription regulation, chromatin regulation	[151]
12	Homeobox protein CDX-2	CDX-2	*CDX2*, *CDX3*	Transcription regulation, developmental protein	[152]
13	Hypoxia-inducible factor 1-alpha	HIF-1-alpha, bHLHe78	*HIF1A*, *BHLHE78*, *MOP1*, *PASD8*	Transcription factor, master regulator of the adaptive response to hypoxia	[153]
14	Myc proto-oncogene protein	c-Myc, bHLHe39	*MYC*, *BHLHE39*	Transcription factor recognising sequence 5′-CAC[GA]TG-3′	[154]
15	Nuclear receptor corepressor 1	NCOR1	*NCOR1*, *KIAA1047*	Transcription regulation	[155]
16	Paired amphipathic helix protein Sin3a	SIN3A	*SIN3A*	Transcription regulation	[22, 150, 156]
17	Polycomb protein EED	EED, hEED, WAIT-1	*WAIT*-*1*	Transcription regulation, chromatin regulation	[157]
18	Signal transducer and activator of transcription 1-alpha/beta	–	*STAT1*	Transcription factor	[147]
19	Transcription activator MYB	c-Myb	*MYB*	Transcription regulation	[158]
20	Transcription factor AP-1	c-Jun, AP1, p39	*JUN*	Transcription regulation	[159]
21	Transcription factor SOX-2	SOX2	*SOX2*	Transcription regulation	[160, 161]
22	Transcription initiation factor TFIID subunit 1	p250, TAF(II)250, TAFII-250, TAFII250	*TAF1*	Transcription regulation, cell cycle	[150]
Others					
23	DNA-directed RNA polymerase II subunit RPB1	POLR2A, RNAPII	*RNAPII*, *POLR2*	RNA polymerase, transcription	[53, 162]
24	Protein arginine *N*-methyltransferase 5	PRMT5, SKB1 homolog, SKB1Hs	*PRMT5*, *HRMT1L5*, *IBP72*, *JBP1*, *SKB1*	Arginine methyltransferase	[156]
25	Proto-oncogene c-Fos	c-Fos	*FOS*, *G0S7*	DNA-binding	[159]

### BRM aberrations in human cancer

**Fig. 3:**
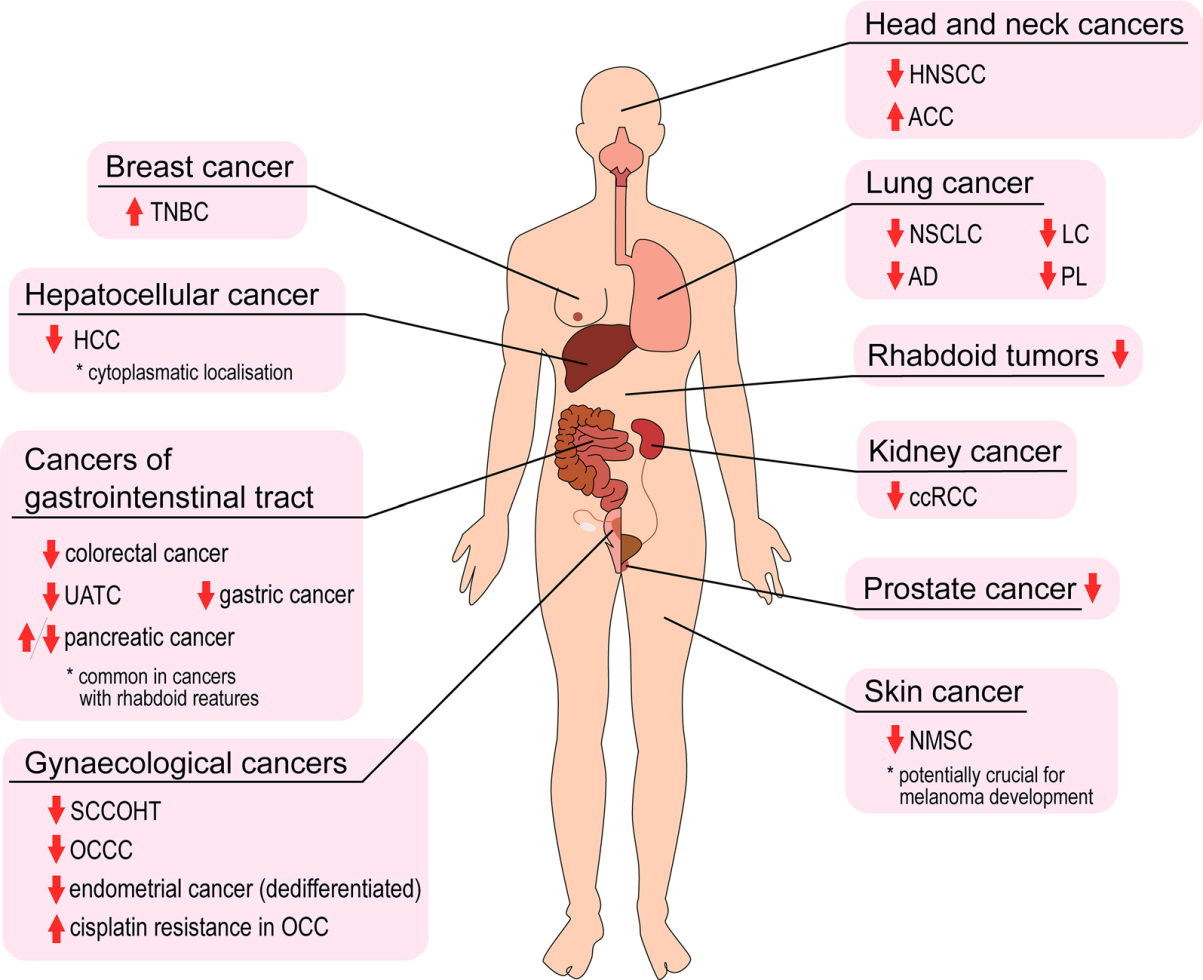
Schematic summary of BRM role in cancer development. ↑—upregulation; ↓—downregulation; *TNBC* triple-negative breast cancer, *HCC* hepatocellular carcinoma, *UATC* upper aerodigestive tract, *SCCOHT* small cell carcinoma of the ovary, hypercalcaemic type, *OCCC* ovarian clear cell carcinoma; *OCC* ovarian cell carcinoma, *HNSCC* head and neck squamous cell carcinoma, *ACC* adenoid cystic carcinoma; *NSCLC* non-small cell lung cancer; *AD* adenocarcinoma od the lung, *LC* large cell carcinoma of the lung; *PL* pleomorphic carcinoma of the lung; *ccRCC* clear cell renal cell carcinoma, *NMSC* non-melanoma skin cancer

The importance of an altered level and/or aberrant function of BRM in various cancers is not fully understood, although there is abundant evidence indicating the crucial BRM role in carcinogenesis. About 15% of all cancers display numerous aberrations in BRM abundance or impairment, that may lead to cancer development or progression (Fig. 3).

#### Rhabdoid tumours

Malignant rhabdoid tumour (MRT) is an extremely aggressive type of cancer that affects mostly children. The mutations in *SMARCB1* an INI1/SNF5/BAF47-encoding gene were found in this type of cancer, suggesting the mutation in a gene encoding core SNF5-type subunit of SWI/SNF CRCs as a driving mutation for this cancer type [61, 62]. This INI1 alteration was accompanied by BRM epigenetic silencing in about 70% of MRT cases. BRM was silenced by the HDAC-driven mechanism or by *SMARCA2* promoter polymorphisms [53]. Interestingly, BRM expression was induced by INI1 re-expression and synthetic flavonoid treatment. BRM re-expression was necessary for flavonoid or INI1 re-expression induced growth inhibition of rhabdoid cell line [63].

#### Lung cancer

The downregulation of BRM is most frequently reported in lung cancer (LC) especially in non-small cell lung carcinoma (NSCLC). In case of the adenocarcinoma (AD), the amount of cases with BRM depletion is 6–17% [64, 65], although in the group of poorly differentiated ADs the fraction of cases with BRM depletion reaches 92% [64–66]. The highest downregulation of BRM protein was observed in the pleomorphic carcinoma of the lung (PL)—over 40% cases [66], and in the large cell carcinoma of the lung (LCCL)—33–50% cases [64, 66]. BRM staining is positive in lepidic growth components in LC and becomes significantly lower or negative in invasive parts [65, 66].

The BRM level in the primary NSCLC is associated with the survival rate. The overall survival for the group of patients with loss of nuclear BRM was significantly lower comparing to patients with high BRM level. Additionally, a membrane form of BRM was observed in immunohistochemistry in some samples. In the case of AD, 5-year survival of patients with positive staining for the membrane BRM was significantly lower than in case of patients without the membrane BRM, suggesting that the presence of the membrane BRM form may be a good prognostic marker [67]. However, the function of the membrane form of BRM remains unknown and there are no data regarding such localisation of other subunits of SWI/SNF CRCs. Intriguingly, loss of BRM and BRG1 in this type of cancer is correlated with loss of tumour cells’ ability to differentiate [31, 65]. A correlation between loss of BRG1 and BRM and epithelial–mesenchymal transition (EMT) in lung cancer was reported, especially in poorly differentiated ADs. Loss of BRM protein was more frequent in heavy smokers, supporting the hypothesis that BRM depletion enhances susceptibility to cancer induced by the carcinogen exposure [65, 68]. On the other hand, targeting BRM in the BRG1-deficient lung cancer (NSCLC) sensitised cancer cells (cell lines) to radiotherapy [69].

#### Renal cell carcinoma

SWI/SNF CRCs are aberrant in clear cell renal cell carcinoma (ccRCC), the most common type of renal cancer [70, 71]. The 3% of all ccRCC cases were BRM-negative. All BRM-negative cases were classified as poorly differentiated grade 4 tumours [72]. It is also significant that loss of BRM was observed only in ccRCC, and not in other types of renal cancers like fumarate hydratase-deficient RCC [73].

Interestingly, in the case of RCC, the same pattern as in lung cancer was observed—poorly differentiated tumours or poorly differentiated parts of tumours were lacking a BRM subunit, while more differentiated areas showed BRM expression [72, 74] suggesting that alterations of *SMARCA2* gene occur during de-differentiation of ccRCC. Thus, BRM loss may be specifically linked to the tumour aggressiveness. In BRM-deficient ccRCC tumours almost 90% cases displayed genetic alterations in the *SMARCA2* gene, such as mutations, promoter methylation or chromosomal aberrations. Interestingly, the alterations were very rare in low-grade part of analysed tumours and in non-neoplastic tissue [74]. These observations strongly suggest that loss of BRM occurred during cancer progression.

#### Cancers of gastrointestinal tract

Loss of SWI/SNF subunits correlates with undifferentiated tumour phenotypes in gastrointestinal tract (GI) cancers. Interestingly, in rare GI cancers, with rhabdoid features no concurrent loss of BRM and BRG1 ATPases was observed. On the other hand, concomitant inactivation of BRM and INI1/SNF5/BAF47 frequently lost in rhabdoid carcinomas was reported [75].

Inactivation of the BRM-encoding *SMARCA2* gene by the presence of promoter indel polymorphisms correlates with higher risk of colorectal cancer [76]. These promoter indels increase risk of upper aerodigestive tract cancers (UATC) more than twofold suggesting that BRM downregulation may be significant for development and progression of GI cancers [77].

The reduction of BRM expression was observed in gastric cancers, while in such cases, BRG1 level was unaffected [78]. Importantly, this effect was characteristic only for cancer cells, but was not seen in premalignant lesions suggesting the importance of BRM loss at later stages of the stomach cancer development [79]. In about 10% of gastric cancers, methylation of *SMARCA2* promoter region was identified [79].

Altered levels of BRM expression are also observed during pancreatic cancer development, although the mechanism is not yet fully understood. High levels of BRM are associated with patients’ poor survival, linked to larger tumour size, metastasis to other organs, lymphatic invasion and stage IV disease [80]. On the other hand, the downregulation of BRM may be a significant marker in the pancreatic cancer [57], indicating that BRM levels undergo dynamic changes in different stages of the disease. BRM silencing in pancreatic cancer cell line correlates with lower cell viability, proliferation rate and growth both in vitro and in vivo. This fact may be contrasted with the observation that downregulation of BRM is vital for pancreatic cancer progression [57] indicating that BRM is actually essential for this process. Intriguingly, in data presented by Shain et al., no pancreatic cancer cell lines with decreased BRM expression were observed [81]. A recent study indicated that BRM promotes pancreatic cancer growth and chemoresistance via activation JAK2/STAT3 pathway [57]. It also transcriptionally regulates the miR-302a-3p and promotes pancreatic cancer metastasis by epigenetic modulation of SOCS5/STAT3 signalling axis [56]. However, to the large extent, the role of BRM in pancreatic cancer remains elusive.

#### Hepatocellular cancer

According to current knowledge, normal human hepatocytes display negative staining for BRG1 and positive for BRM protein [82]. The lack of BRG1 protein in normal human hepatocytes is, however, counterintuitive to expectations, especially given that in mouse models Brg1 is more important than Brm for liver regeneration after injury [28]. By contrast, a majority of hepatocellular carcinoma (HCC) is characterised by the positive BRG1 staining. In 22.5% of HCC cases, the loss of BRM protein was found while in 15% of analysed cases both BRM and BRG1 were lost. The depletion of BRM in HCC significantly corresponded to poor overall survival [82]. Pasic et al. correlated this phenomenon with the occurrence of BRM promoter polymorphisms that were also found in other cancers, leading to poorer patient survival [83].

Interestingly, in HCC tumour cells, additional cytoplasmic localisation of BRM was found, while no such cases were observed in healthy hepatocytes. This indicates that changes in BRM localisation may also contribute to carcinogenesis, although the mechanism of this phenomenon remains unknown [82].

#### Head and neck cancers

The head and neck squamous cell carcinoma (HNSCC) is one of the most common head and neck cancers, accounting for up to 90% of cases. In 16% of patient samples, the total loss of BRM was demonstrated, 11% featured weak staining and 16% were mosaic. Occurrence of *SMARCA2* promoter region polymorphisms correlates with HNSCC risk, especially in HPV-positive oropharyngeal cancer [84].

In 5% of cases of salivary gland adenoid cystic carcinoma (ACC), the *SMARCA2* mutation was found [85]. Additionally, both mRNA and protein level of BRM were significantly elevated in ACC cells, comparing to the healthy tissue. Interestingly, the BRM overexpression was observed in every sample in all tumour areas, although ACC is the most heterogeneous cancer type. This strongly suggests that salivary gland ACC is characterised by BRM overexpression [86] although the specific mechanism of BRM action in the ACC cells needs further elucidation.

#### Breast cancer

Cohet et al. described that the presence of both SWI/SNF ATPases is crucial for optimal cell cycle progression in non-malignant mammary epithelial cells and knock-down of either BRM or BRG1 affects cell cycle, while the double knock-down of BRM and BRG1 results in cell death [87].

The analyses of a set of different breast cancers with various subtypes and stages revealed the increase of BRM levels in a significant majority of analysed cases [88]. However, the authors did not mention whether nuclear, cytoplasmic or other BRM staining was taken into consideration. Additionally, it was observed that both BRG1 and BRM are required for the triple-negative breast cancer (TNBC) proliferation and that double *SMARCA2* and *SMARCA4* knock-down results in slowed tumour growth in xenografts [88]. By contrast, a statistically significant downregulation of *SMARCA2* transcript was observed in all breast cancer types comparing to healthy tissue. Based on this observation, it was postulated that expression of *SMARCA2* and *SMARCA4* has prognostic value [89], although another study shows that the BRM protein level varies among various breast cancer types. Namely, decreased level of BRM was observed in MDA-MB-231 (TNBC cancer cell line) comparing to the less malignant MCF-7 (ER positive) cells. Furthermore, BRM regulates tight junction protein expression via targeting their promoters, thus takes part in the breast cancer metastasis [90].

#### Gynaecological cancers

Although in gynaecological cancers many alterations of SWI/SNF subunits were observed [91–94], differences in BRM expression were found only in small cell carcinoma of the ovary, hypercalcaemic type (SCCOHT), ovarian cell carcinoma (OCC) and in the endometrial cancer.

SCCOHT is a rare subtype of the ovarian cancer that affects mainly young women. At first, loss of BRG1 protein caused by somatic and germline mutations in *SMARCA4* gene (coding for BRG1 protein) in SCCOHT was identified by a few groups [95–99]. Recently, loss of BRM protein was found in this type of cancer [100, 101]. Importantly, no mutations in *SMARCA2* gene were found, suggesting epigenetic control of *SMARCA2* gene expression.

Noteworthy, for SCCOHT analysis, the ovarian clear cell carcinoma (OCCC), a different type of ovarian cancer, was used as a basis for comparison. Interestingly, in OCCC tumours, the lack of BRM or BRG1 was found but never loss of both ATPases. The mutations in the *SMARCA2* gene, in about 2% of OCCC samples, were described [102]. Although, in epithelial ovarian cancer (the most common type of ovarian cancer) BRM overexpression strongly correlates with resistance to cisplatin, probably due to the reduction of apoptosis and influence of metabolism and cancer-associated signalling pathways [103]. These findings confirmed earlier discoveries that the downregulation of BRM increases cisplatin sensitivity [104].

The lack of both ATPases also was found in endometrial cancer [105], although no information about clinical significance of such loss has been so far described.

#### Prostate cancer

Analysis of prostate cancer samples revealed that level of BRM was significantly downregulated in primary prostate tumours and metastases, although BRG1 level increased with the disease progression [106, 107]. The slightly lower signal for BRM was found in malignant sites comparing to the non-invasive parts of cancer [107]. BRM has been found to be required for the proliferation of AR-dependent prostatic adenocarcinoma cells [58].

#### Skin cancers

Alterations in BRM expression seem to be important for the non-melanoma skin cancer (NMSC) development, and in this case, the role of *SMARCA2* as a susceptibility gene is strongly pronouncing. Analyses of NMSC patient samples, specifically squamous cell carcinoma (SCC) and basal cell carcinoma (BCC) with comparison to benign precancerous lesions—actinic keratosis (AK), which is claimed to be a progenitor for SCC and BCC development, and normal skin showed that the transcript level of *SMARCA2* gene decreased in SCC cells. By contrast, no differences were observed for *SMARCA4* gene [108]. Surprisingly, at the protein level, both BRM and BRG1 were downregulated about tenfold in both SCC and BCC comparing to AK and normal skin, indicating altered regulation of their expression through some other mechanism. The authors suggested that loss of ATPases happens after the development of benign skin lesions, where no downregulation of BRM was observed [108]. Additionally, a mutation in *SMARCA2* gene was identified in 17% of NMSC. Since this mutation was observed only in SCCs and BCCs and not in precancerous lesions or normal skin, this observation suggested that the mutation was preferentially selected in the process of cancer development [109]. Subsequently, a possible mechanism underlying BRM loss in NMSC was discovered based on a mouse model. It was found that mouse keratinocytes with deleted *Brm* (*Brm*^−/−^) grew faster than normal (*Brm*^+/+^) after UV-irradiation. This suggests the ability of Brm null mutant keratinocytes to escape UV-induced cell cycle arrest faster than in normal cells. Moreover, although *Brm*^−/−^ keratinocytes accumulate more DNA damage, they do not compensate for this with more intense DNA repair [110]. All these effects result in the ability of *Brm* null mutant keratinocytes to undergo selective pressure that can cause overgrowth of cells with accumulated mutations over normal cells and hence lead to cancer development [110].

SWI/SNF ATPases are also claimed to be important components in regulation of microphthalmia-associated transcription factor (MITF) expression. MITF is a key factor linked to development of melanoma. BRG1 was described as a main epigenetic regulator of *MITF* expression. However, in case of cancers with inactivated BRG1, BRM replaces siblings ATPase and performs their function. In such cases, pharmacological exclusion of BRM could lead to the reduction of melanoma growth or even cancer cell death [111].

#### Other neoplasms

In case of leukaemia, evidence of the importance of BRM protein is rather weak. In this cancer type, SWI/SNF complexes are mainly built around the BRG1 ATPase that is essential for survival and growth of this neoplasm [112]. BRM is the main ATPase expressed in quiescent hematopoietic stem cells suggesting that loss of BRM significance takes place at the time of leukaemia development. This hypothesis is supported by discovery of Doménech et al. [113] who identified mutations in *SMARCA2* gene in leukemic cells. In acute myeloid leukaemia with monosomy, 7 (about 13% of cases) novel mutations of *SMARCA2* gene were identified, although these are not thought to be driving mutations because the samples were collected at more advanced stages of carcinogenesis [114]. In proximal-type epithelioid sarcoma with pure rhabdoid tumour features, additional loss of BRM was found [115]. Additionally, the occurrence of SNPs in the *SMARCA2* gene seems to correlate with risk for oligodendroglioma development [116].

Collectively, in various types of cancer, loss or strong decrease of BRG1 or BRM and sometimes both ATPases, was observed (Table 2). BRG1 and BRM seem to have similar/redundant function in cancer cells, although, they play different roles during human development. This hypothesis supports the observation that mutation in BRG1 encoding gene is frequently associated with CSS while the mutation in BRM-encoding gene is more typical for NCBRS. These two different genetic disorders carry some similar phenotypic aberration but also differ from each other (see Fig. 1). The exact role of BRM and BRG1 in somatic cells and how the imbalance in their abundance trigger to disorders or cancer development still remains unknown. Therefore, investigation of BRM and BRG1 differential function in various cancer types and in somatic cells seems to be one of the most exciting and important directions for further research.

**Table 2:** The alterations of BRM and BRG1 in various cancers

Cancer type	*SMARCA2*/BRM		Refs.	*SMARCA4*/BRG1		Refs.
	Protein expression	Genetic alterations		Protein expression	Genetic alterations	
Rhabdoid tumours	↓ (concomitant INI1 loss)	Epigenetic silencing	[63, 121]	↓	Epigenetic silencing	[121, 163–165]
Lung cancer	↓	Promoter polymorphism	[64–67]	↓/(loss)	Mutations (LOF)	[31, 64, 166, 167]
Renal cell carcinoma (RCC)	↓	Mutations	[72–74]	Concomitant loss with INI1 in rhabdoid subtype	n/d	[168]
Gastric cancer	↓	n/d	[78, 79]	Normal/↑	n/d	[78, 169]
Pancreatic cancer	↑/↓	n/d	[57, 80, 81]	↑?	n/d	[80]
Hepatocellular cancer	↓	Promoter polymorphism	[82, 83]	↑	n/d	[82]
HNSCC	↓	n/d	[84]	n/d	n/d	–
ACC	↑	n/d	[86]	Normal	n/d	[86]
Oral cancer	n/d	n/d	–	↑	Not determined	[170]
Breast cancer	↑/↓	Not determined	[88, 89]	↑	2%	[88, 171]
SCCOHT	↓(loss)	n/d	[100, 101]	↓/(loss)	Somatic and germline mutations	[95–99]
OCCC	↓/↑^a^	Mutations	[102]	n/d	n/d	–
Endometrial cancer	↓	Mutations	[105]	↓	Mutations	[105]
Prostate cancer	↓	n/d	[106, 107]	↑	n/d	[106, 107]
Melanoma	n/d	n/d	–	↑	Rare mutations	[172, 173]
NMSC	↓	Mutations	[108]	↓	n/d	[108]

n/d—no data

^a^Upregulation of BRM in OCCC corresponds to resistance to cisplatin [103, 104]

### Mechanism of BRM alteration in cancer

Alterations of BRM were reported in various cancer types, but only in some of them, *SMARCA2* mutations were found. Therefore, it is highly probable that various mechanisms of BRM control exist including mutation-independent regulation of *SMARCA2* gene expression.

#### Mutations

In the majority of cancers lacking BRM, no mutations of the *SMARCA2* gene were found suggesting that epigenetic regulation plays more crucial role in the BRM inactivation [82, 117, 118]. However, *SMARCA2* mutations were found in 78.2% of BRM-deficient ccRCC cases, although about half of them were silent [74]. All detected mutations were specific for cancerous tissue, especially low differentiated, and none or very low-level mutations were found in BRM-positive tissues as well as adjacent non-malignant tissues [74]. *SMARCA2* gene mutations were also identified in about 10% of gastric cancers [79]. In NMSC, a mutation resulting in substitution of glutamine by lysine was discovered. Interestingly, this mutation type—G:C to T:A substitution is observed after UV-irradiation, what is a usual cause of skin cancer [109]. *SMARCA2* mutations of unknown effect were also found in leukaemia [113].

#### Epigenetic modifications

Methylation of CpG sites on *SMARCA2* promoter region is the key mechanism of BRM alterations [74]. It was recently found that Polycomb repressive complex 2 (PRC2) causes epigenetic suppression of *SMARCA2*, by inducing H3K27me3 silencing pattern on its promoter region [119].

In BRM, deficient ccRCC methylation refers to over 40% of cases and was found only in low-differentiated tumour areas [74]. A similarly observation was made in the AD of the lung, where *SMARCA2* promoter methylation correlated with poor prognosis [40]. Nonetheless, in some cancers with lack/low BRM level, no mutations and no hypermethylation were found in the *SMARCA2 locus* [108] suggesting the existence of other mechanisms involved in the control of BRM expression or protein stability.

Additionally, the activity of BRM is modulated by posttranslational modifications, for example acetylation can lead to BRM inactivation [120]. Three acetylation sites were identified in the BRM protein, although their specific functions remain elusive, and whether acetylation of only one or all sites is required for BRM inactivation is unknown [121].

#### Promoter insertion polymorphisms

Insertions in the promoter sequence of *SMARCA2* gene (at positions −741 and −1321) was specified as silencing-type polymorphism, leading to development of many types of cancer [122]. Interestingly, the promoter insertions cause HDACs recruitment and result in *SMARCA2* gene silencing [68]. Such insertions were associated with higher risk of lung cancer [68], colorectal cancer [76], and head and neck squamous cell carcinoma [84]. Increased risk of developing upper aerodigestive tract cancers appears only in case of double homozygous variants of such polymorphisms [77]. In the case of pancreatic cancer, the presence of *SMARCA2* promoter polymorphisms is associated with poor prognosis for patients with diagnosed cancer rather than specific cancer risk [123].

#### Chromosomal aberrations

The loss of chromosome 9p, the *SMARCA2* gene location, results in BRM loss and enhanced cancer aggressiveness. In ccRCC aberrations of chromosome, 9p (monosomy or deletion) was found in over 40% of analysed BRM-deficient tumours [74]. Currently, the loss of 9p chromosome is used as a prognostic marker for ccRCC [124].

Moreover, loss of the BRM subunit may occur in cells with multiplication of chromosome 9. Multiple abnormalities also may lead to simultaneous silencing of all copies of the gene, e.g. by mutation and CpG methylation that occur in the same cancer cell [74].

### BRM overexpression

Although a few malignancies with the upregulation of BRM were identified, a specific mechanism leading to BRM overexpression in neoplasms was not identified. In ACC samples, BRM was elevated on both protein and transcript level [86]. Overexpression of BRM correlated with poor survival and chemoresistance in pancreatic cancer. Similarly, in ovarian cancer, high level of BRM promoted resistance to cisplatin [80, 103].

### New treatment strategies in BRM-altered cancers

Only some malignancies with overexpression of BRM protein were identified, whilst in most of the neoplasms, the BRM is strongly downregulated or lost. The restoration of BRM protein in BRM-deficient cancers leads to impeded cancer cell growth [63]. This effect may be obtained with chemical compounds. Thus, the search of such compounds is encouraged [118, 125].

The first study considering direct BRM targeting was development of selective SMARCA2/4 bromodomain inhibitor (PFI-3), although PFI-3 did not reveal antiproliferative effect in cancer cells. This observation indicated that bromodomain is not a proper therapeutic target, and research focused on targeting helicase/ATPase domain in BRM for synthetic-lethality therapy [126]. Recently, small molecules for inhibition of BRM and BRG1 ATPase activity were discovered. In a BRG1-deficient lung cancer xenograft model, these inhibitors downregulated BRM-dependent gene expression and exhibited antiproliferative effect upon oral administration [127]. Another approach based on proteolysis-targeting chimera (PROTAC) has been designed to target BRM/BRG1 subunits of SWI/SNF CRCs employing a bromodomain ligand to recruit the VHL, the E3 ubiquitin ligase facilitating protein degradation [128].

Polymorphisms in *SMARCA2* gene in position −747 and −1321 are suggested to be responsible for HDAC recruitment, and HDAC inhibitors (HDACi) cause upregulation of both BRM transcript and protein levels in cell lines [26, 100, 120, 129, 130]. Application of HDACi in patient groups seems to be a promising therapy, especially now as HDACi are approved by Food and Drug Administration and European Medicines Agency, and are becoming a more popular choice of treatment in various types of human cancer [131]. A similar effect on BRM expression was obtained during flavopiridol (synthetic flavonoid) treatment of rhabdoid cell lines [63]. Importantly, some studies demonstrated that whilst utilisation of HDACi effectively induces BRM, it also leads to an increase of BRM acetylation which impairs its function, giving no therapeutic effect overall [120].

In tumours with *SMARCA2* depletion caused by PRC2-driven methylation, usage of EZH2 inhibitors seems to be a promising therapy. Effectiveness of this kind of drug was demonstrated for ovarian cancer (SCCOHT) in in vitro models [132]. Interestingly, in SCCOHT, a synergistic effect was observed, when EZH2 inhibitors were used together with HDACi [133]. Unfortunately, such an approach appeared to be not suitable for every type of cancer with BRM depletion: for instance, in lung cancer cell lines and pancreatic cancer cell lines, no therapeutic effect was observed.

BRM appeared as an attractive therapeutic target and induction of its activity may be helpful in cancer treatment. Therefore, a special reporter system was designed to identify novel compounds that restore not only BRM protein level, but also its function, giving hope for effective epigenetically focused treatment [125].

BRM and BRG1 are mutually exclusive ATPase subunits of SWI/SNF CRCs. Therefore, BRM targeting in BRG1-deficient cancer is expected to cause synthetic lethality [134]. So far, this phenomenon has been described for lung and ovarian cancers [126, 127, 135].

**Fig. 4:**
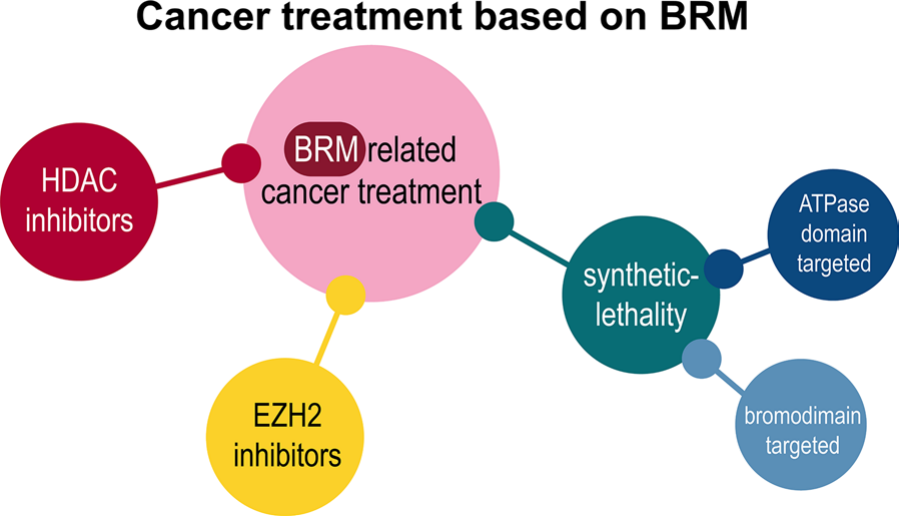
Cancer treatment related to BRM protein. Ideas for utilising BRM in anticancer therapy are emerging. Such therapies, taken currently into consideration, are based on BRM level restoration by, e.g. HDAC inhibitors and E2F inhibitors. A very promising but demanding idea is based on a synthetic lethality approach, targeted against BRM ATPase domain or bromodomain

Possibilities for incorporation of BRM/SMARCA2-related targeted therapy into the clinic are still developing and new strategies seems to be very promising and effective (Fig. 4).

## Conclusions

BRM deficiency or downregulation was found in various types of cancer, although its function in cancer development and progression remains elusive. Interestingly, the mutation rate in *SMARCA2* gene is quite rare compared to mutations in *SMARCA4*, suggesting a less important role of BRM ATPase containing SWI/SNF CRCs in carcinogenesis. Some data indicated *SMARCA2*/BRM as a tumour suppressor or tumour susceptibility gene, whereas overexpression of BRM caused cancer resistance for chemotherapy leading to cancer progression indicating its promoting role. All available data concerning BRM in cancer suggest that BRM function differs depending on cancer type. Thus, BRM acts in some cases as a tumour suppressor and in other cancer types or stages as a tumour or disease-promoting factor. Moreover, in some tumour types, loss or downregulation of BRM occurs during cancer development in late stage or in poorly differentiated/undifferentiated cancer cells suggesting clonal selection of BRM-deficient cancer cells. Additionally, some *SMARCA2* missense mutations result not only in BRM loss of function but also may lead to production of gain-of-function BRM protein, likely influencing the whole BRM containing SWI/SNF CRC’s activity.

Based on ample recently published data, BRM seems to be a perfect target for various anticancer therapies including ATPase activity inhibition, synthetic lethality induction, etc. However, in view of current knowledge, i.e. given the high similarity of the ATPase domain among various proteins involved in the control of numerous regulatory processes, such optimism should be moderated by the risk of severe side effects of such therapies through off-target effects. During designing of the BRM-based anticancer therapy, another important fact needs to be urgently taken into consideration, namely, the observation that normal hepatocytes are negative for BRG1 ATPase. In such case, any BRM-targeting small molecule drugs can cause severe and unexpected negative effects on liver activity which may be impossible to detect during initial tests on cancer lines or in mice, as in this model, Brm seems to have a diverse function than in human liver. The damaging effect of BRM-targeting compounds likely may be observed in any tissue characterised by the lack of BRG1 protein. Therefore, additional study on BRM function in normal tissues and cancers is clearly required for better understanding of the interdependence between both ATPases, to precisely and safely treat cancer patients with new anti-BRM compounds or compound-based therapies.

Another important issue in the study of BRM function in development and carcinogenesis is the alternative splicing of *SMARCA2* gene, which is relatively unexplored. According to the NCBI database, there are seven alternative transcripts of *SMARCA2* gene. A similar situation is observed in mice, where six alternative transcripts exist. There are no data about the tissue or developmental stage-specific expression of these splicing variants either in mice or in humans. The relevance of BRM alternative splice variants in cancer is overlooked, although such a multiplicity of alternative BRM splicing variants suggests far more potential regulatory or pathological functions of the BRM protein which may be specific for certain cancer types or developmental stages. Knowledge about the existence of truncated BRM forms in cancer is very limited, although it is very likely that such incomplete but still partially functional proteins are produced due to unusual splicing events frequently occurring in various cancers. Such truncated BRM forms may have a very strong negative or gain-of-function effect on the functionality of the whole SWI/SNF complex, and thus may lead to the de-regulation of numerous important regulatory cellular processes fine-tuned by SWI/SNF complexes.

## Data Availability

Not applicable.

## Abbreviations

ACC: adenoid cystic carcinoma
AD: adenocarcinoma of the lung
ATP: adenosine tri-phosphate
CRC: chromatin-remodelling complex
ccRCC: clear cell renal cell carcinoma
CSS: Coffin–Siris syndrome
E2F: E2 promoter binding factor
EMT: epithelial–mesenchymal transition
GI: gastrointestinal tract
HCC: hepatocellular carcinoma
HDAC: histone deacetylase
HDACi: histone deacetylase inhibitors
HNSCC: head and neck squamous cell carcinoma
IHC: immunohistochemistry
LC: lung cancer
LCCL: large cell carcinoma of the lung
MITF: microphthalmia-associated transcription factor
NCBRS: Nicolaides–Baraitser syndrome
NMSC: non-melanoma skin cancer
NSCLC: non-small cell lung cancer
OCC: ovarian cell carcinoma
OCCC: ovarian clear cell carcinoma
PL: pleomorphic carcinoma of the lung
PRC2: polycomb repressive complex 2
Rb: retinoblastoma protein
RCC: renal cell carcinoma
SCC: squamous cell carcinoma
SCCOHT: small cell carcinoma of the ovary, hypercalcaemic type
SNP: single-nucleotide polymorphism
SWI/SNF: switch/sucrose non-fermenting
UATC: upper aerodigestive tract cancers
